# Influences on intentions for obstetric practice among family physicians and residents in Canada: an explorative qualitative inquiry

**DOI:** 10.1186/s12884-022-05165-1

**Published:** 2022-11-19

**Authors:** Emily Gard Marshall, Kathleen Horrey, Lauren R. Moritz, Richard Buote, Agnes Grudniewicz, Laurie J. Goldsmith, Ellen Randall, Lori Jones, M Ruth Lavergne

**Affiliations:** 1 Primary Care Research Unit, Department of Family Medicine, Dalhousie University, 1465 Brenton Street, Suite 402, Halifax, NS B3J 3T4 Canada; 2 Department of Family Medicine, Dalhousie University, Halifax, Canada; 3 Telfer School of Management, University of Ottawa, Ottawa, Canada; 4 Faculty of Health Sciences, Family Medicine and Primary Care, Simon Fraser University, Burnaby, Canada; 5 Lee Kong Chian School of Medicine, Nanyang Technological University, Novena, Singapore; 6 School of Population and Public Health, University of British Columbia, Vancouver, Canada; 7 Department of History, University of Ottawa, Ottawa, Canada

**Keywords:** Family practice, Obstetrics, Scope of practice, Socio-ecological models

## Abstract

**Background:**

Many family medicine residency graduates indicate a desire to provide obstetric care, but a low proportion of family physicians (FPs) provide obstetric care within their practice. This suggests personal preference alone may not account for the low proportion of FPs who ultimately provide full obstetric care. If decisionmakers plan to augment the number of FPs providing obstetric care, barriers to the provision of such care must first be identified. Within this paper, we explore the perspectives of both family practice residents and early-career FPs on the factors that shaped their decision to provide obstetric care.

**Methods:**

In this qualitative study, we analyzed a subset of interview data from three Canadian provinces: British Columbia, Ontario, and Nova Scotia (*n* = 18 family practice residents; *n* = 39 early-career FPs). We used thematic analysis to analyze data relevant to obstetric care practice, applying the socio-ecological model and comparing themes across participant types, gender, and province.

**Results:**

Participants described influences affecting their decision about providing obstetric care. Key influencing factors aligned with the levels of the socio-ecological model of public policy (i.e., liability), community (i.e., community needs), organizational (e.g., obstetric care trade-offs, working in teams, sufficient exposure in training), interpersonal practice preferences (i.e., impact on family life, negative interactions with other healthcare professionals), and individual factors (i.e., defining comprehensive care as “everything but obstetrics”). Many participants were interested in providing obstetric care within their practice but did not provide such care. Participants’ decision-making around providing or not providing obstetric care included considerations of personal preferences and outside influences.

**Conclusions:**

Individual-level factors alone do not account for the decrease in the type and amount of obstetric care offered by FPs. Instead, FPs’ choice to provide or not provide obstetric care is influenced by factors at higher levels of the socio-ecological model. Policymakers who want to encourage obstetric practice by FPs should implement interventions at the public policy, community, organizational, interpersonal, and individual levels.

**Supplementary Information:**

The online version contains supplementary material available at 10.1186/s12884-022-05165-1.

## Background

Family physicians (FPs) are trained to offer comprehensive healthcare across the life course [1]. Although family medicine scope of practice can include full obstetric care (prenatal, intrapartum/deliveries, and postpartum), many FPs in North America do not provide intrapartum care/deliveries as part of their practice [1–7]. Numerous factors shape FPs’ decision to provide obstetric care, including an interest in obstetrics early in medical training [3], concerns about irregular hours and work/life balance [1, 2, 5, 8], fear of poor patient outcomes [1, 2], and burnout [9]. Many family medicine residency graduates indicate a desire to provide obstetric care in their practice, demonstrating that personal preference alone may not account for the low proportion of FPs who ultimately provide full obstetric care [1, 10].

A low proportion of FPs providing full obstetric care may be cause for concern, particularly in rural regions where many patients rely on FPs for obstetric care [1, 2, 4]. Additionally, the provision of obstetric care has been posited to influence burnout among FPs: its inclusion as part of the broad scope of practice is associated with lower levels of burnout [11]. However, in what is referred to as a family medicine-obstetrics paradox, among early-career FPs, including full obstetrics in practice has been associated both with risk factors that increase burnout (e.g., stress and call schedules) and with features that lessen burnout (e.g., enjoyment and variety in practice) [12]. Addressing barriers to the provision of obstetric care among FPs may help to protect them against burnout.

In this paper, we present the perspectives of both family practice residents and early-career FPs (< 10 years in practice) on factors influencing their decision to provide full obstetric care or not. Our study explored viewpoints from three Canadian provinces (British Columbia [BC], Ontario [ON], and Nova Scotia [NS]), providing a unique cross-provincial outlook across the early stages of FP careers when most FPs make the decision to provide obstetric care as part of their practice [13]. We apply a socio-ecological model [14] to help elucidate intervention opportunities if policymakers seek to augment the provision of obstetric care.

## Methods

### Study design and participants

We analyzed semi-structured qualitative interview data from an exploratory, cross-provincial study exploring practice intentions and decision-making among residents and early-career FPs (< 10 years in practice). The full details of this study are available in the published protocol [15]. Participants in BC, ON, and NS were recruited via provincial medical association newsletters, family medicine residency programme email lists, and social media (Twitter and Facebook). Participants were eligible to participate in the study if they were a family medicine resident, or if they were a FP who had completed their family medicine residency between 2008 and 2018 and were currently practicing. Participants also had to be practicing in BC, ON, or NS. Potential study participants completed a demographic screening questionnaire to ensure they met the study’s inclusion criteria and facilitate diverse purposeful sampling based upon previously identified characteristics (i.e., gender, relationship status, whether they have dependents, rurality, specialization, practice/training location, practice type/model) [15]. During the recruitment period, 359 residents and family physicians completed the demographic screening questionnaire. Of these, 32 family medicine residents and 69 early-career FPs were invited to participate in the study. Participants were chosen based upon responses to the questionnaire to ensure maximum variation. Interviews were completed with 31 of 32 family medicine residents and 63 of 69 early-career FPs invited to participate across the three Canadian provinces (NS, ON, BC). Seven interviews were declined due to scheduling conflicts, lack of response, or an undisclosed reason. Participants were offered an honorarium.

### Data collection and analysis

Using semi-structured interview guides (Additional file 1), telephone interviews (~ one hour) with family medicine residents and early-career FPs explored factors shaping their practice intentions and choices [15]. To reduce the possibility of recall bias, interviewers used the same interview guide, and used probes to elicit rich responses. Interviews were conducted by three Master’s or Ph.D. trained qualitative research staff members under the supervision of experienced PhD university faculty and the principal investigators of this study (EGM, AG, LG, & RL). Interviews were audio-recorded and transcribed verbatim. Interviewers recorded interview summaries and reflections following each interview. Recruitment continued until no new themes were identified within the interviews. Data were analyzed iteratively through an inductive thematic analysis [16] and then inductively coded using codebooks developed with early interview transcripts by the three research staff members. Codebooks were refined iteratively to incorporate emerging themes. Study data were managed using NVivo software [17].

Data analysis for this paper involved thematic analysis of text relevant to obstetric practice pulled from thematic coding. In this paper, “obstetrics” typically refers to the FP practice of labour and delivery services, although participants may have referred to other aspects of obstetric care when using this term [12]. The team met to discuss themes until all were confident that participant perspectives were represented. Data were observed by gender and career stage (i.e., resident or early-career FP) and subgroup analyses were performed across these characteristics. Themes were organized according to a socio-ecological model [14] to identify specific factors influencing the decision to provide obstetric care at the level of the individual, interpersonal, organization, community, and public policy. Socio-ecological models have been used in healthcare research [18–21] and enable researchers to identify predictive factors beyond the individual level alone, situating the factors where improvements through policy and educational efforts could be directed. This study was approved by the Simon Fraser University (#H18-03291), University of Ottawa (#S-05–18-776), and Nova Scotia Health Authority research ethics boards (#1023561). Informed consent was obtained from all participants and all methods were carried out in accordance with relevant guidelines and regulations.

## Results

Eighteen family medicine residents and 39 early-career FPs discussed obstetric practice during their interviews. Most participants who spoke about obstetrics practiced in NS. The sample included similar numbers of men and women with variations in relationship status and caring responsibilities who practiced in various settings and models (Table 1).

**Table 1:** Demographic characteristics of participating family practice residents (*n* = 18) and early-career family physicians (*n* = 39) who discussed obstetrics

		**Family practice residents (*n* = 18)**	**Early-career family physicians (*n* = 39)**
**Province**	Nova Scotia	9 (50.0%)	19 (48.7%)
	Ontario	8 (44.4%)	11 (28.2%)
	British Columbia	1 (5.6%)	10 (25.6%)
**Dependents**	Yes, child(ren)	4 (22.2%)	25 (64.1%)
	Yes, adult(s)	1 (5.6%)	1 (2.6%)
	Yes, both	0 (0.0%)	1 (2.6%)
	No	13 (72.2%)	12 (30.8%)
**Gender**	Man	9 (50.0%)	13 (33.3%)
	Woman	8 (44.4%)	26 (66.7%)
	Unreported	1 (5.6%)	0 (0.0%)
	Other	0 (0.0%)	0 (0.0%)
**Practice Setting**^**a**^	Inner city	6 (33.3%)	8 (20.5%)
	Urban/suburban	13 (72.2%)	23 (59.0%)
	Small town	3 (16.7)	10 (25.6%)
	Rural	7 (38.9%)	15 (38.5%)
	Remote	2 (11.1%)	3 (7.7%)
**Current Year of Residency**	PGY1	9 (50.0%)	***N/A***
	PGY2	9 (50.0%)	***N/A***
	No data	0 (0.0%)	***N/A***
**Last Year of Residency**	2010–2012	***N/A***	6 (15.4%)
	2013–2015	***N/A***	15 (38.5%)
	2016–2018	***N/A***	18 (2.6%)
**Medical School Graduation Location**	Canada	14 (77.8%)	29 (74.4%)
	Outside of Canada	4 (22.2%)	10 (25.6%)
	Unreported	0 (0.0%)	0 (0.0%)
**Payment Type**^**a**^	Blended	***N/A***	3 (7.7%)
	Capitation	***N/A***	3 (7.7%)
	Fee-for-service	***N/A***	29 (74.4%)
	Salary	***N/A***	13 (33.3%)
	Service contract	***N/A***	4 (10.3%)
	Sessional or per diem or hourly	***N/A***	14 (35.9%)
	Other	***N/A***	1 (2.6%)
**Practice Model**^**a**^	Group	13 (72.2%)	27 (69.2%)
	Interprofessional team	12 (66.7%)	14 (35.9%)
	Solo	3 (16.7%)	7 (17.9%)
	Other	0 (0.0%)	8 (20.5%)
**Practice Type After Residency**	Comprehensive	8 (44.4%)	28 (71.8%)
	Focused	6 (33.3%)	8 (20.5%)
	Special interest	4 (22.2%)	6 (15.4%)
	Other	0 (0.0%)	1 (2.6%)
**Relationship Status**	Single/divorced/separated/widowed	9 (50.0%)	14 (35.9%)
	Married/common-law/life partner	9 (50.0%)	25 (64.1%)
	Unreported	0 (0.0%)	0 (0.0%)

^a^ Some totals higher than sample size due to participants working in multiple locations/falling into multiple categories

Themes were identified at each of the socio-ecological model levels: 1) public policy factors (i.e., concerns about liability and risk in providing obstetric care); 2) community-level factors (i.e., community needs); 3) organizational factors (i.e., workload and practice variety considerations, call groups or teams as an enabler, influence of training and exposure to obstetric care, costs associated with sufficient training, gender-based barriers); 4) interpersonal factors (i.e., negative interactions with other healthcare professionals, impact on personal and family life); and 5) individual factors (i.e., defining comprehensive care as “everything but obstetrics”).

### Public policy factors

#### Concerns about liability and risk in obstetric care

As a participant explained, deliveries’ riskiness is reflected in higher insurance costs for FPs who practice obstetrics. The perceived riskiness is described within the quotes from FPs and residents. As some expressed, it was “terrifying” to provide care related to labour and delivery, and exposure to high-risk situations discouraged them from providing obstetric care, even if they previously may have liked to. As an FP expressed, the higher fees and stress and the fact that *“outcomes aren’t always favourable”* may discourage FPs from providing obstetric care (Table 2).

**Table 2:** Illustrative participant quotes on concerns about liability and risk in obstetric care

*… obstetrics is higher risk. So, as family physicians … we pay liability insurance through [CMPA]. And for any family physician that practices obstetrics, you actually pay higher insurance fees because it’s a higher risk practice of medicine than straightforward general family medicine … there are … things that can happen that are quite stressful, and outcomes aren’t always favourable. And people may not want to take on that responsibility or risk.* (Family Physician, woman, NS)
*Yeah, at one point I did consider maybe doing some obstetrical work as well. But I then realized that actually I hate that. I hate obstetrics. It was stressful … if I had like pursued that further and then later realized actually I don’t want to wake up at 3:00 in the morning and have someone potentially bleed out on me … That’s like not exciting for me. That’s terrifying.* (Family Physician, woman, ON)
*… it’s the only time in medicine that you’ve got two healthy beings that can either remain healthy or something bad can happen. And when that happens, it’s devastating. But if someone comes into emerg with a heart attack, if they die, it’s sad. But they had a heart attack. If you save them, everybody’s elated and happy. You’re seeing sick patients and you’re trying to make them better. OB’s terrifying because you’ve got healthy patients and the expectation is they stay healthy*. (Family Physician, woman, BC)
*… I’ve seen very critical situations. And I realized that I would not want to be put in that position. Because obstetrics is particularly high risk in my opinion for family doctors to be doing… by just seeing a couple of like very high-risk situations that almost ended very badly kind of just reinforced the fact that that’s not an area of practice that I want to be involved in.* (Resident, man, NS)

### Community-level factors

#### Community needs as a driver of what services to offer

FPs who would otherwise have provided obstetric care often did not end up doing so if their community had other needs, such as medical assistance in dying (MaiD), or if their patient population was primarily elderly. FPs also considered the services available from other healthcare providers within the community, explaining that if enough providers already offered obstetric care, they were less likely to do so themselves.

Interviewees described the needs of the communities where they practice as a driver of providing obstetrics care or not. FPs may be the sole primary care provider and feel obligated to offer as many services as they can to meet community needs, which may or may not include obstetrics. Participants described greater opportunity to offer obstetric care in rural communities compared to urban communities where such care is often shared with other obstetric providers (Table 3).

**Table 3:** Illustrative participant quotes on community needs as a driver of what services to offer

*… I really like obstetrics … But* *MaiD* *was something that was under-serviced. And the woman that was only doing it at the time was quite stressed out. And when she approached me, it just made sense to do it. So, I think it’s what my community needs definitely influences what I would change my practice to.* (Family Physician, woman, BC)
*I think that just the nature of the work itself, it’s more elderly people. We don’t see a lot of pediatrics or obstetrics.* (Family Physician, woman, ON)
*Well, I don’t do any obstetrics… Yeah, probably obstetrics would be the biggest thing [excluded from practice]. Like I guess theoretically I could deliver a baby but it’s not too likely. Most places I work, they have obs.* (Family Physician, man, NS)
*I worked with … a husband and wife family physician couple in a small town in [Canadian province]. And they both had comprehensive family practices but just due to the nature of like it was a town of 5,000 people so they had to take on other roles. So, one of them also did anesthesia, and another one had a pretty extensive OB practice.* (Resident, unreported gender, NS)
*… I think that’s based a lot on the community and also the size of the community, and what’s already set up. Because … my friends who have all graduated in different sized communities, in a smaller place, a lot of them will do obstetrics. And in the big urban centres, they just don’t.* (Family Physician, woman, BC)

### Organizational factors

Organizational factors can include influences within one’s work or educational settings. We identified five influencing factors at this level: considerations of disruption, workload, and variety within one’s practice; enabled by working in call groups or teams; influence of sufficient training and exposure to obstetrics; financial and opportunity costs of being sufficiently trained; and “gendered expectations” discouraged men from providing obstetrics.

#### Considerations of disruption, workload, and variety within one’s practice

FPs and residents shared concerns about the potential disruption to their practice due to the workload and unpredictable time commitment associated with deliveries as a deterrent to practice. However, a few FPs and residents described wanting variety in their practice and were thus motivated to offer obstetric care (Table 4).

**Table 4:** Illustrative participant quotes on considerations of disruption, workload, and variety within one’s practice

*…if there was a patient that was in labour, I was kind of following them. So, I was working quite a bit. And I know it’s a bit different as a resident. But the physicians here, like they do follow their patients. And you don’t know when your patient is going to deliver. So, you’re pretty much on call all the time. It can be disruptive to your office.* (Resident, woman, NS)
*Towards October of last year, I also started doing more prenatal clinic on top of that. And by I want to say February of this year, I found that I was just really, really overloaded. Working like insanely too much. Probably 60, 80-h weeks routinely.* (Family Physician, man, NS)
*… I’m now in the process of trying to find out what I want to do when I graduate… I do know that I want some diversity in my practice. So, I want a little bit of clinic time but then I also want time outside of the clinic … Right now, I’m sort of leaning towards either primary care obstetrics or emerg.* (Resident, woman, NS)

#### Enabled by working in call groups or teams

Among FPs and residents, sharing work amongst a call group or team was an enabler to providing obstetric care. One FP explained that, in some communities, it is difficult to participate in obstetrics and avoid burnout without a call group or team to support obstetric work. A resident explained that the availability of a good call group model was a key consideration of where to practice. However, the organization and sustainability of call groups can be challenging, potentially leading to burnout if providers are on-call too long or have nobody else to whom they can refer patients. This theme also relates to interpersonal factors, as participants described that they “trust” other providers who may deliver their patients’ babies (Table 5).

**Table 5:** Illustrative participant quotes on working in call groups or teams as an enabler

*I have a really awesome call group… if I want to take the weekend off and have a weekend with my family, and not have to worry about being pulled away… I can sign out to them… I have a group of physicians that will care for my patients. … I trust that my patients are in really good hands… my patients know that I’m part of a call group… they all know that there are going to be times that I can’t show up for their delivery… they all kind of seem … to be understanding of that reality.* (Family Physician, woman, NS)
*Well, I feel very lucky to be part of a clinic that is very supportive of one another. So, within the clinic, there are now 5 of us who have our own practices but all cover each other because we all are only in the clinic a few days a week. We share all of the obstetrics patients because we all typically do a day of call a week. So therefore don’t commit ourselves to one particular patient for their entire pregnancy. Like being available for them. So, we’re in a call group.* (Family Physician, woman, BC)
*I live in a community that …is just under 100,000, maybe 80,000…the obstetrics doctors… have their own sort of system and their own call and their own website. And you’d have to almost join that, I think, at this point if you were doing obstetrics.* (Family Physician, woman, BC)
*There are other provinces or other places that had different sort of models that would suit my lifestyle a bit better. And I think I’m speaking more to the obstetrics part of it. Because I kind of mentioned that like here like you’re basically on the clock. They do have a bit of a call schedule here, but it’s not as well established as in other communities. So, I’m definitely looking at different provinces. And we’re even looking at the States as well.* (Resident, woman, NS)
*I do prenatal care. I would have liked to keep doing deliveries but there’s no other family docs in [city] right now that do it. So, I would be on call for my own patients, which deterred me from pursuing that.* (Family Physician, woman, NS)

#### The influence of sufficient training and exposure to obstetrics

FPs and residents felt that adequate exposure to obstetrics during their training was influential in their decision to provide obstetric care. Alternatively, having insufficient exposure to obstetrics during training and fewer opportunities to build the skills and confidence needed to provide obstetric care discouraged many interviewees from providing such care. Having negative experiences during training was also discouraging. Some FPs described the importance of continued exposure to obstetrics during early-career practice opportunities, including through locum opportunities. FPs expressed the challenge of returning to providing obstetric care after time away, concerned about the loss of skills (Table 6).

**Table 6:** Illustrative participant quotes on the influence of sufficient training and exposure to obstetrics

"*Given the time I’ve put into being able to do obstetrics, I’ve delivered way less babies than I think is reasonable. And so, I don’t have those skills. And so, I don’t do it anymore. It absolutely has been a problem*." (Family Physician, man, ON)
*Time in residency is a big factor… I was thinking of doing obstetrics but I’m not sure if I feel comfortable. Like based on my two months I had … I don’t know if I got enough deliveries. So, I have to look at some elective time. And I’d have to go away for that most likely. Which would normally be fine but like with a family, it’s a bit more challenging.* (Resident, woman, NS)
*… we had lots of opportunities to do obstetrics during our residency. So that was wonderful. Because I know that not all residents feel ready to practice obstetrics, especially in a place like here where it’s rural, where you have to like figure things out at night… it’s not like a great place for a brand-new person just because you have to be really independent and kind of confident in your skills… I was lucky in my residency training, we did do a lot of obstetrics. And that was sort of an expected skill set to have even if you didn’t plan on doing obstetrics.* (Family Physician, woman, NS)
*…in second year medical school… one of the things that you could do… was called Deliver a Baby. You could actually sign up and spend a night in the hospital working alongside a family doctor and help deliver a baby… it felt like an adrenaline rush in the most profound way for me following a woman through her entire labour and being present and witnessing the family doctor deliver the baby, and put the baby on the mom’s chest, and watching the emotion of the mother and her partner… I remember getting goosebumps … I went home the next morning, and I was invigorated that whatever I did as a career, it was going to involve that.* (Family Physician, woman, NS)
*I was actually planning on doing my third-year fellowship in obstetrics… I ended up not doing it because I had my daughter, and I didn’t want to be away from home… instead of that I chose to do a locum with family medicine with family medicine obstetrics support. I used that to further develop my skills in obstetrics during my locum time…now I have my practice, and… I’m part of a family medicine obstetrics group. And I also do emerg in like a small community where I trained. I did a rural family medicine program. So, like during our training, we did emerg cover and obstetrics and all that. So, I wanted to continue to maintain those skills. And so, I still do that.* (Family Physician, woman, ON)
*I think that like there’s different skill sets in a way. I think it’s always easier to move from acute care to like less acute care… it’s always a choice later on. But as I do less and less of… pediatrics and obstetrics, it becomes more difficult to go back into.* (Family Physician, woman, ON)

#### Financial and opportunity costs of being sufficiently trained

Several FPs and residents considered the additional time and costs needed to get sufficient training as influencers of their decision not to provide obstetric care. Although they would have liked to offer such care, interviewees described wanting to enter the workforce or achieve personal goals rather than taking extra time needed for training (Table 7).

**Table 7:** Illustrative participant quotes on financial and opportunity costs of being sufficiently trained

*If I don’t feel at the end of my two years that I’m comfortable with delivering babies or I’m not comfortable doing emerg then I won’t be providing those services … I know there’s options of doing an extra… 3 months or a year depending on the route you take… those are things that I definitely considered. However, it comes down to… time. Like being in residency a bit longer … thinking about that can be difficult to comprehend. And then also too like financially… we have quite a bit of debt. So, me spending another three months or a year in residency versus me working, that… comes into play as well.* (Resident, woman, NS)
*So, the reason I don’t deliver babies … is I didn’t get enough experience, I feel. I had 20 shifts on obstetrics, and only delivered 6 or 7 babies. So, you know, that shaped me in the opposite direction. I really had wanted to provide that as a service, but I just don’t have the experience. And I didn’t want to take an extra… 6 months or a year, to do more training.* (Family Physician, woman, ON)
*So, when I finished my two-year family medicine training, I actually went on to do a fellowship in women’s health and obstetrics. Those were areas of interest of mine that I wanted to gain more focused clinical skills around.* (Family Physician, woman, NS)

#### “Gendered expectations” discouraged men from providing obstetrics

Some participants who identified as men described how gendered expectations moderated their exposure to obstetrics, ultimately discouraging them from providing obstetric care. They described an “anti-male” culture in educational spaces, assumptions that they would not be interested, patient preference or cultural safety, or feeling they could not empathize with birthing patients. An FP who identified as a woman described a positive experience with a male physician and the need for more men in the field (Table 8).

**Table 8:** Illustrative participant quotes on “gendered expectations” discouraging men from providing obstetrics

*…I think there’s a very strong* … *almost an anti-male involvement as a medical student element of obstetrical floors and on birthing units. Which I think I saw less once I was a resident but was still present. So, I guess that experience with more obstetrics than I necessarily wanted actually cemented my desire not to do obstetrics.* (Resident, man, NS)
*… you wouldn’t get called for things [because of identifying as a man]. People would assume that I wasn’t interested. I worked with one… family doctor who did her own deliveries. And she didn’t call me for the first few deliveries because she thought I wasn’t truly interested in it because I was a guy. And only believed me when I showed up sick as a dog and said yes, please call me, I would like to do it, this is why I’m here. I’ve had a lot of trouble with that.* (Family Physician, man, ON)
*… it was very hard trying to get involved in obstetrics as a male. There’s a lot of institutional inertia against men in that. Or I felt that there was.* (Family Physician, man, ON)
*I think where it impacts me most, the fact that I’m male… is with prenatal and obstetrics simply because … women ask me questions about pregnancy and delivery and their bodies and such, and I can give them the academic and my own sort of experiential point of view, but I can’t truly empathize with what they’re going through… I think that puts me at a disadvantage and I think it affects my credibility a little bit in terms of talking through it with them… that bothers me but there’s nothing I can do about that. But I’m very aware of that.* (Family Physician, man, NS)
*…we did have a male physician, and we thoroughly enjoyed that. It kind of balanced things out. Because in obstetrics, as of lately, we’ve had mostly female obstetricians. We have all female staff, and all female midwives, and all female family practitioners. So, it’s nice to keep things a little even… we would love to have more males in our area of work. But that’s just not the case right now.* (Family Physician, woman, NS)

### Interpersonal-level factors

The interpersonal level includes interactions with other individuals. Two themes were identified at this level – negative interaction with other healthcare providers and impact on personal and family life.

#### Negative interactions with other healthcare providers

A few FPs described how negative interactions in obstetric care during training or early practice discouraged them from providing obstetric care. An FP explained how working in “toxic” workplaces solidified their decision not to provide obstetric care (Table 9).

**Table 9:** Illustrative participant quotes on negative interactions with other healthcare providers

*…I ended up having … several bad experiences with the obstetrician who was on call… it just kind of put me over the edge and said, look, this is…I’m not enjoying this, I’m not having fun. I don’t want to get treated like I’m being treated. So, I’m not going to do this anymore.* (Family Physician, man, NS)
*…we ended up going to the operating room five times out of the seven deliveries for postpartum hemorrhage. Which is a crazy amount of time[s]. I don’t know why, it was just a really bad run. And [the obstetrician] basically … ended up being quite short with me… I felt like I was being treated … disrespectfully… I just said … I know that I’m new but I’m still a staff … He basically told me, “I don’t want to talk to you. I want to talk [to] the nurses.” Which was a bit insulting. Like I said, I know I’m new but I’m still a staff physician. I have been trained. Telling me to get off the phone and put a nurse on is a little bit belittling.* (Family Physician, man, NS)
*… some of the people that I worked with… There was one attending specifically, he was just really, really intense and made me think, yeah, this is not for me.* (Family Physician, woman, ON)
*… during residency, we did do quite a lot… I probably did 3 or 4 months straight of obstetrics. But even in that experience, there was a lot of conflict with the allied health providers that made it a really kind of toxic atmosphere. So almost none of the residents wanted to do it… I think of our class of 22 residents, maybe only 3 or 4 are actually doing obstetrics because of that… it was not a very good learning environment.* (Family Physician, woman, BC)

#### Impact on personal and family life

Impact on family life was a commonly cited reason not to provide obstetric care, although only mentioned by interviewees who identified as women. Major challenges discussed by participants included arranging childcare, being away from their children, and coordinating schedules with a partner. Several participants felt that the unpredictable hours associated with labour and delivery discouraged them from providing obstetric care (Table 10).

**Table 10:** Illustrative participant quotes on the impact on personal and family life

*…when doing obstetrics… in the soft call system where you can be called any time from home, you really need to have all of the supports available… to be able to drop off kids at a minute’s notice.* (Family Physician, woman, ON)
*… when I finished residency, I always thought I’d do obstetrics. But then I had these twins, and it’s been a lot… they’re four now and I always thought when they get to kindergarten, perhaps I can go back to obstetrics because I love it. But it just hasn’t worked for my family.* (Family Physician, woman, BC)
*…I wanted to be home for dinner with my family every night… the way I grew up… having dinner with my family every night, that was a priority. I didn’t want to be stuck in the hospital every weekend or every evening… I think that really shaped how I practice.* (Family Physician, woman, NS)
*I would love to. It’s certainly like my all-time favourite thing to do in family medicine. But my husband is an obstetrician. And realistically balancing two call schedules with family… would be a bit of a nightmare.* (Family Physician, woman, NS)

### Individual factors

#### Defining comprehensive care as “everything but obstetrics”

Although obstetric care is part of family medicine training, several FPs and residents did not consider it a necessary component of their definition of comprehensive care. Many participants offered, or planned to offer, comprehensive services but commonly noted that they described comprehensive care as “everything but obstetrics.” Among those respondents who did not plan to offer delivery services, some planned to offer prenatal care for pregnant patients up to around 20 weeks and then care for newborns and pediatric patients (Table 11).

**Table 11:** Illustrative participant quotes on defining comprehensive care as “everything but obstetrics”

*[I offer] full-service family medicine. Everything except obstetrics after 20 weeks.* (Family Physician, man, BC)
*Ideally it would be a comprehensive family medicine, with the exception of obstetrical care… that would include pediatrics, adolescent and sexual health, care for female and male adults, and geriatrics as well… with the exception of prenatal care past 20 weeks, and obstetrical deliveries.* (Resident, man, NS)
*…I’d like to do like a full service, even have like some permission to do some emerg work as well. So probably outside of the city, like a little bit outside of the city at full service. Everything but obstetrics.* (Resident, woman, ON)

## Discussion

Among our family medicine resident and early-career FP participants, interest in providing obstetric care varied – some expressed interest in providing obstetric care, while others expressed disinterest. Many participants were interested in providing obstetric care but did not provide it. Participants’ decision-making around providing or not providing obstetric care included considerations of personal preferences and outside influences. Factors that influenced decisions to provide or not to provide obstetric care have been contextualized within the socio-ecological model.

**Fig. 1:**
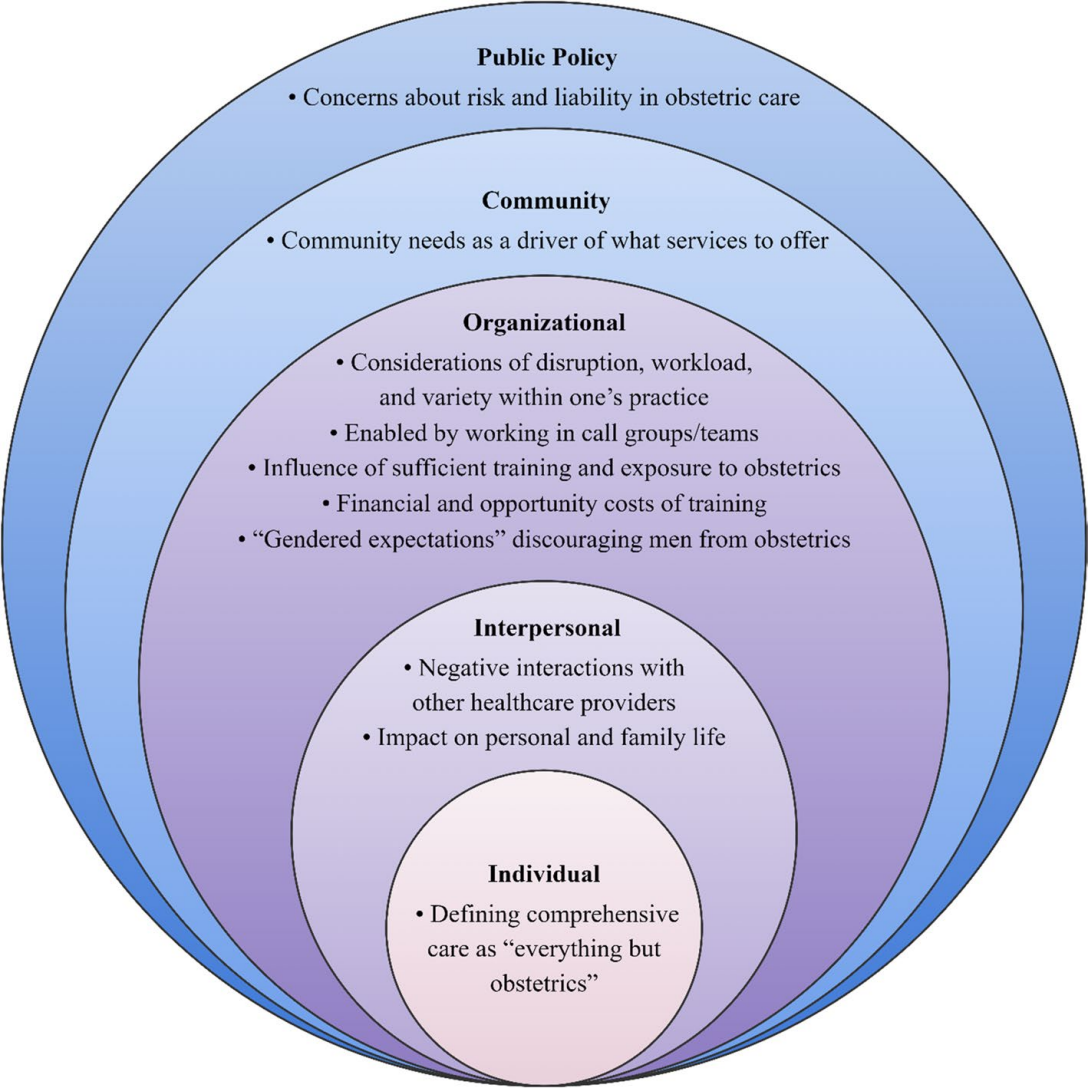
The socio-ecological barriers and enablers to offering intrapartum care

Through this study, factors influencing the decision to provide obstetric care were identified at all five socio-ecological levels: public policy, community, organizational, interpersonal, and individual (Fig. 1) [14]. Public policy influences encompass regulatory policies and system-level decisions. Our analysis identified that concerns about liability and risk influenced decision-making about whether to provide obstetric care. Community-level factors include the community’s needs and the availability and distribution of resources within a defined region. FPs may be more likely to provide obstetric care in rural regions and less so in urban regions where other providers are available, or different services are needed. Organizational influences encompass characteristics of educational and work environments. Participants perceived that obstetric care could disrupt regular practice, but working in call groups could be an enabler to providing obstetrics. Interviewees suggested that sufficient exposure during training encouraged obstetrics provision and continued exposure allowed FPs to maintain necessary skills to provide obstetric care. Providers could be discouraged from providing obstetric care by the cost of receiving adequate training and negative “gendered expectations” of trainees who identified as men. Interpersonal-level factors include social relationships. Factors at this level included negative interactions with other healthcare providers and the impact that the provision of obstetric care could have on one’s personal and family life. Finally, individual-level factors include personal beliefs, which may be impacted by other levels of the ecological model. Most participants intended to offer a comprehensive practice yet felt that a comprehensive practice encompassed “everything but obstetrics,” a perception that may be shaped by higher-level influences, such as concerns about risk and community need.

In North America, fewer FPs are providing obstetric care [2, 5–7]. Results from our study indicate that individual preference alone may not account for this decline, as many participants in our study wished to provide obstetric care but were discouraged from doing so by several systemic factors. As McLeroy and colleagues argue, system-level problems require system-level solutions [14]. If system planners wish to increase the number of FPs providing obstetric care, interventions should address these systemic challenges. Public policy deterrents could be mitigated by addressing provider concerns about liability and risk in obstetric care and associated costs [7, 22, 23]. At the community level, community needs will continue to drive service offerings; it may be valuable in rural areas to have greater interprofessional, collaborative team support for FPs who wish to provide obstetric care [2, 7, 23]. Additional organizational interventions include increasing exposure to obstetrics during training, creating supportive cultures within training institutions, and improving acceptance of men who wish to provide obstetric care [24]. Collaborative working arrangements such as care teams and obstetrics on-call groups could facilitate obstetric practice [2, 7]. Working with supportive colleagues could reduce the impact of providing obstetric care on one’s practice and family life by sharing the responsibilities to meet the community’s needs [6, 7, 22]. Formal team arrangements can also reduce interpersonal difficulties between obstetric providers [25].

### Strengths and limitations of the study

This study provides rich insights into the factors influencing family medicine residents’ and early-career FPs’ decision to provide obstetric care or not. While we conducted interviews with a substantial number of participants, recruitment was purposeful and participants self-identified interest in participating; thus, their views are not generalizable to the population of all family medicine residents and FPs. Around half of the participants who spoke about obstetrics were from the province of Nova Scotia, which may also affect generalizability to FPs working in other jurisdictions. As with all qualitative research, the experiences and perspectives of the researchers played a role in the identification and interpretation of findings. The team includes a variety of skilled personnel, including FPs, family medicine educators, health services researchers, and experienced PhD-trained qualitative researchers who worked to ensure participant perspectives were accurately represented and the research was performed in a rigorous and credible way.

Future work could explore whether the concepts identified within our analyses are generalizable to the population (e.g., through a survey of all family medicine residents and early-career FPs). Such research would inform the development of policies and interventions that may remove barriers to the provision of obstetric care for those FPs who wish to provide such care.

## Conclusions

Our objective was to present the perspectives of family practice residents and early-career FPs concerning factors influencing their decision to provide obstetric care. Using a socio-ecological model, influencing factors were identified at the public policy, community, organizational, interpersonal, and individual levels. Our results demonstrate that the decision to provide obstetric care is not influenced solely by individual factors. Instead, barriers at all levels of the socio-ecological model must be addressed if a goal is to enable more FPs to provide obstetric care.

## Supplementary Information

**Additional file 1.**

## Data Availability

Participants of this study did not agree for their data to be shared publicly, so supporting data are not available.

## Abbreviations

FP: Family Physician
BC: British Columbia
ON: Ontario
NS: Nova Scotia
MAiD: Medical Assistance in Dying
OB: Obstetrics

## References

[1] Hansen ER, Eden AR, Peterson LE (2019). A qualitative study of trainee experiences in family medicine-obstetrics fellowships. Birth.

[2] Hedden L, Munro S, McGrail KM, Law MR, Bourgeault IL, Barer ML (2019). Is attending birth dying out?: trends in obstetric care provision among primary care physicians in British Columbia. Can Fam Physician.

[3] Bédard MJ, Berthiaume S, Beaulieu MD, Leclerc C (2006). Factors influencing the decision to practise obstetrics among Québec medical students: a survey. J Obstet Gynaecol Can.

[4] Pinto M, Rochat R, Hennink M, Zertuche AD, Spelke B (2016). Bridging the gaps in obstetric care: perspectives of service delivery providers on challenges and core components of care in rural Georgia. Matern Child Health J.

[5] Koppula S, Brown JB, Jordan JM (2014). Teaching primary care obstetrics: insights and recruitment recommendations from family physicians. Can Fam Physician.

[6] Nesbitt TS, Kahn NB, Tanji JL, Scherger JE (1992). Factors influencing family physicians to continue providing obstetric care. West J Med.

[7] Tong ST, Makaroff LA, Xierali IM, Puffer JC, Newton WP, Bazemore AW (2013). Family physicians in the maternity care workforce: Factors influencing declining trends. Matern Child Health J.

[8] Leigh JP, Tancredi DJ, Kravitz RL (2009). Physician career satisfaction within specialties. BMC Health Serv Res.

[9] Dyrbye LN, Varkey P, Boone SL, Satele DV, Sloan JA, Shanafelt TD (2013). Physician satisfaction and burnout at different career stages. Mayo Clin Proc.

[10] Barreto TW, Eden AR, Hansen ER, Peterson LE (2018). Barriers faced by family medicine graduates interested in performing obstetric deliveries. J Am Board Fam Med.

[11] Weidner AKH, Phillips RLJ, Fang B, Peterson LE (2018). Burnout and scope of practice in new family physicians. Ann Fam Med.

[12] Barreto TW, Eden A, Brock A (2020). The impact of practicing obstetrics on burnout among early-career family physicians. Fam Med.

[13] Dove M, Dogba MJ, Rodríguez C (2017). Exploring family physicians’ reasons to continue or discontinue providing intrapartum care: qualitative descriptive study. Can Fam Physician.

[14] McLeroy KR, Bibeau D, Steckler A, Glanz K (1988). An ecological perspective on health promotion programs. Health Educ Q.

[15] Lavergne MR, Goldsmith LJ, Grudniewicz A, Rudoler D, Marshall EG, Ahuja M (2019). Practice patterns among early-career primary care (ECPC) physicians and workforce planning implications: protocol for a mixed methods study. BMJ Open..

[16] Braun V, Clarke V (2006). Using thematic analysis in psychology. Qual Res Psychol.

[17] QSR International Pty Ltd. NVivo (Version 12). 2018 [cited 2021 Nov 2]. https://www.qsrinternational.com/nvivo-qualitative-data-analysis-software/home?_ga=2.176207653.1677916018.1635851483-1731282329.1635851483 . Available from

[18] Hennein R, Mew EJ, Lowe SR (2021). Socio-ecological predictors of mental health outcomes among healthcare workers during the COVID-19 pandemic in the United States. PLoS ONE.

[19] Mengesha ZB, Perz J, Dune T, Ussher J (2017). Refugee and migrant women’s engagement with sexual and reproductive health care in Australia: a socio-ecological analysis of health care professional perspectives. PLoS ONE.

[20] Harris Walker G, Gonzalez-Guarda R, Yang Q, Shah S, Prvu BJ (2021). Socio-ecological perspective on factors influencing acute recovery of younger stroke survivors: a mixed methods study. J Adv Nurs.

[21] Litchfield I, Perryman K, Avery A, Campbell S, Gill P, Greenfield S (2021). From policy to patient: Using a socio-ecological framework to explore the factors influencing safe practice in UK primary care. Soc Sci Med.

[22] Rayburn WF, Petterson SM, Phillips RL (2014). Trends in family physicians performing deliveries, 2003–2010. Birth.

[23] Dresden GM, Baldwin LM, Andrilla CHA, Skillman SM, Benedetti TJ (2008). Influence of obstetric practice on workload and practice patterns of family physicians and obstetrician-gynecologists. Ann Fam Med.

[24] Craig LB, Buery-Joyner SD, Bliss S, Everett EN, Forstein DA, Graziano SC (2018). To the point: gender differences in the obstetrics and gynecology clerkship. Am J Obstet Gynecol.

[25] Brown D, Brewster C, Karides M, Lukas L (2011). The phenomenon of collaboration: a phenomenologic study of collaboration between family medicine and obstetrics and gynecology departments at an academic medical center. Qual Rep.

